# Failure Analysis of Ultra-High Molecular Weight Polyethylene Tibial Insert in Total Knee Arthroplasty

**DOI:** 10.3390/ma15207102

**Published:** 2022-10-13

**Authors:** Veronica Manescu (Paltanea), Iulian Antoniac, Aurora Antoniac, Gheorghe Paltanea, Marian Miculescu, Ana-Iulia Bita, Stefan Laptoiu, Marius Niculescu, Alexandru Stere, Costel Paun, Mihai Bogdan Cristea

**Affiliations:** 1 Faculty of Material Science and Engineering, University Politehnica of Bucharest, 313 Splaiul Independentei, District 6, 060042 Bucharest, Romania; 2 Faculty of Electrical Engineering, University Politehnica of Bucharest, 313 Splaiul Independentei, District 6, 060042 Bucharest, Romania; 3 Academy of Romanian Scientists, 54 Splaiul Independentei, 050094 Bucharest, Romania; 4 Faculty of Medicine, Titu Maiorescu University, 67A Gheorghe Petrascu Street, 031593 Bucharest, Romania; 5 Department of Orthopedics and Trauma I, Colentina Clinical Hospital, 19-21 Soseaua Stefan cel Mare, 020125 Bucharest, Romania; 6 Medical Ortovit Ltd., 8 Miron Costin Street, 011098 Bucharest, Romania; 7 National Institute for Research and Development in Microtechnologies IMT-Bucharest, 126A Erou Iancu Nicolae Street, 077190 Bucharest, Romania; 8 Department of Morphological Sciences, Carol Davila University of Medicine and Pharmacy, 37 Dionisie Lupu Street, 020021 Bucharest, Romania

**Keywords:** UHMWPE, wear, oxidation process, total knee arthroplasty, implant failure analysis, macrophotography, light stereomiscroscopy, scanning electron microscopy, FTIR analysis, oxidation index, mechanical characterization

## Abstract

Knee osteoarthritis is treated based on total knee arthroplasty (TKA) interventions. The most frequent failure cause identified in surgical practice is due to wear and oxidation processes of the prothesis’ tibial insert. This component is usually manufactured from ultra-high molecular weight polyethylene (UHMWPE). To estimate the clinical complications related to a specific prosthesis design, we investigated four UHMWPE tibial inserts retrieved from patients from Clinical Hospital Colentina, Bucharest, Romania. For the initial analysis of the polyethylene degradation modes, macrophotography was chosen. A light stereomicroscope was used to estimate the structural performance and the implant surface degradation. Scanning electron microscopy confirmed the optical results and fulfilled the computation of the Hood index. The oxidation process in UHMWPE was analyzed based on Fourier-transform infrared spectroscopy (FTIR). The crystallinity degree and the oxidation index were computed in good agreement with the existing standards. Mechanical characterization was conducted based on the small punch test. The elastic modulus, initial peak load, ultimate load, and ultimate displacement were estimated. Based on the aforementioned experimental tests, a variation between 9 and 32 was found in the case of the Hood score. The oxidation index has a value of 1.33 for the reference sample and a maximum of 9.78 for a retrieved sample.

## 1. Introduction

Knee arthroplasty as a surgical intervention is considered an efficient treatment for osteoarthritis. It is a standard procedure in almost all hospitals in Europe and the United States of America (USA). The statistical data collected in the USA report about 1 million surgical interventions performed yearly [1]. In 2010 there were reported 719,000 total knee arthroplasties, of which 10–15% were secondary revision operations. These types of interventions are performed after implant failure. It is expected that in the year 2030, the number of primary TKA will increase, with a percentage of 673%, equal to about 3.48 million procedures.

Regarding the secondary surgical interventions, it is estimated that an increase of 401% between 2022 and 2030 will occur [2]. The main causes of the TKA procedures are the average life expectancy of the population and the high level of physical activity. Unfortunately, these surgical interventions are performed at a younger age than in the past. About 85% of patients reported an excellent post-operator course after a primary TKA [3,4]. Approximately 20% of patients suffered from long-time chronic pain, and supplementary measures and painkillers had to be taken into account [5,6].

The typical knee prosthesis failures are abnormal joint movements, aseptic loosening, infection, periprosthetic fracture, ligament/flexion instability, arthrofibrosis, and material particles’ side effects. In practice, it was concluded that a knee implant works improperly due to multiple causes mentioned above. The revision surgery is a complex procedure, and it is associated with a high risk of infection, complications, prolonged hospitalization time, and a short life expectancy for the secondary prosthesis [7,8].

The main functions of orthopedic knee implants include articular function restauration and pain relief [9,10]. The implant life expectancy depends on the device design and the used biomaterials [11,12]. All the materials must be Food and Drug Administration (FDA) approved or carry the Conformitè Europëenne certificate [13,14].

Usually, the knee implant femoral component, placed at the distal part of the femoral bone, is made from a binary Co-Cr or a ternary Co-Cr-Mo alloy [15]. Oxidized zirconium (OXINIUM) was introduced by Smith + Nephew as an alternative to the Co-based alloys to reduce the polyethylene particle quantity and limit the number of failed prostheses. This material offers a superior advantage because it combines metallic alloy durability with ceramic material biocompatibility, exhibiting a high corrosion resistance [16,17]. The implants made from OXINIUM are characterized by a 15 years life expectancy and a very low revision intervention rate [18,19]. In the case of patients that exhibit nickel allergies, the femoral component can be manufactured from ceramic materials such as alumina (Al_2_O_3_) [20,21], yttria-stabilized zirconia (Y-TZP) [22,23], and zirconia toughened alumina (ZTA) [24]. The ceramic components’ main disadvantage is implant squeezing and reduced mechanical characteristics [25]. Recently, femoral components made from polyetheretherketone (PEEK) received FDA approval due to the good mechanical properties of the material. PEEK has a value of Young’s modulus close to the one of the human bone, so the stress-shielding effect is prohibited [26,27]. By using polymeric materials, the complications of the metallic particles’ emission are avoided. The principal drawback of PEEK is that an important bioinertia characterizes this material. In order to increase its biocompatibility, different solutions, such as surface treatments, are searched [28,29].

The tibial component positioned at the tibial bone proximal part is manufactured from a titanium alloy (Ti6Al4V) [30,31]. This ternary alloy exhibits adequate mechanical properties and can sustain the mechanical stress that appears during the patient gait cycle. Unfortunately, metallic ion emission can be observed in some cases, and allergic, teratogenic, toxic, and carcinogenic effects are evidenced during secondary surgical interventions. Zimmer—Biomet has developed tibial components made from porous tantalum (Ta), denoted as Trabecular Metal^TM^, with excellent mechanical properties, no stress shielding effects, and high biocompatibility [32,33]. Recent studies present alternative materials such as reinforced PEEK with carbon fiber, the so-called PEEK-OPTIMA, and Zeniva ZA-600 CF [34] and concluded that polymeric-based materials could be considered an alternative for titanium alloy in some cases. In the case of old age or overweight patients, UHMWPE tibial components are proposed, and good outcomes are reported because these two categories of people have a limited range of motion and reduced physical outdoor activities [35]. Finally, the tibial component of the BPK-S Integration prosthesis developed by Peter Brehm GmbH, Weisendorf, Germany, was made from BIOLOX^®^ ceramic material [36].

The tibial insert represents the vulnerable part of a knee implant because this component is usually the first that fails since it is made from polymeric materials [37]. The most used material is ultra-high molecular weight polyethylene (UHMWPE), whose properties were improved over time to decrease wear and oxidation [38,39]. Different variants such as highly cross-linked polyethylene (HXLPE) or HXLPE combined with a synthetic analog of vitamin E (alpha-tocopherol) were proposed to prevent the oxidative degradation effect and to reduce the number of failed knees prosthesis due to osteolysis and aseptic loosening [37,40]. UHMWPE is a particular type of polyethylene, being a linear homopolymer, half crystalline with high molecular mass. The material that is used in the orthopedic field has a molecular weight between (3.5–6) × 10^6^ g/mol and a crystallinity grade of about 50–55% [37]. According to the American standard ASTM, UHMWPE must have a molecular weight higher than 3.1 × 10^6^ a.m.u. (atomic mass units), since the standard ISO 11542 (ISO, 2001) recommends a molecular weight of at least 1 × 10^6^ a.m.u [38]. The ultra-high molecular weight polyethylene exhibits three phases. A crystalline lamellar phase, in which the polymeric chains are organized in an orthorhombic crystalline matrix, and an amorphous phase, in which the macromolecules form random wires or act as molecule ties that go out from a crystalline lamella and enter another one. A third phase partial order, which intercalates macromolecules with tight or loose chains, is present in the material. It can be noticed that the crystalline phase has an important contribution to increased mechanical rigidity, while the amorphous phase offers ductility and resilience [39]. When the polyethylene quality is improper, mechanical stresses can result in polymeric particles that induce osteolysis, an acute inflammatory process. It was observed that this phenomenon occurs due to an inadequate sterilization process with gamma radiations. The standards recommend that the sterilization process of the material be carried out in an inert ethylene oxide atmosphere or gas plasma. The cross-linked effect is induced through irradiation, increasing the material wear resistance by a high amount [37]. The most used types of UHMWPE are manufactured by Ticona (Summit, NJ) and are denominated GUR 1020 and GUR 1050 [41]. The GUR 1020 material has an average molecular weight of 3.5 × 10^6^ g/mol, a density of 0.93 g/mL, yield stress higher than 17 MPa, impact strength higher than 210 kJ/m^2^, and an average particle size of 140 μm. Regarding the GUR 1030 type, the material molecular weight is comprised of between (5.5–6) × 10^6^ g/mol. It has a density of 0.93 g/mL, a tensile modulus of 680 MPa, and an impact strength higher than 130 kJ/m^2^ [41]. The tibial insert can also be manufactured from PEEK, showing increased resistance to degradative processes reported in previous cases and good mechanical properties [35].

In some clinical cases, replacing the native human patella with a dedicated part is necessary. There were proposed different solutions, such as a combination between a metallic base and a thin polyethylene surface, but major failure reports were found in the literature. Today, the worldwide accepted solution uses a UHMWPE component [42].

**Figure 1:**
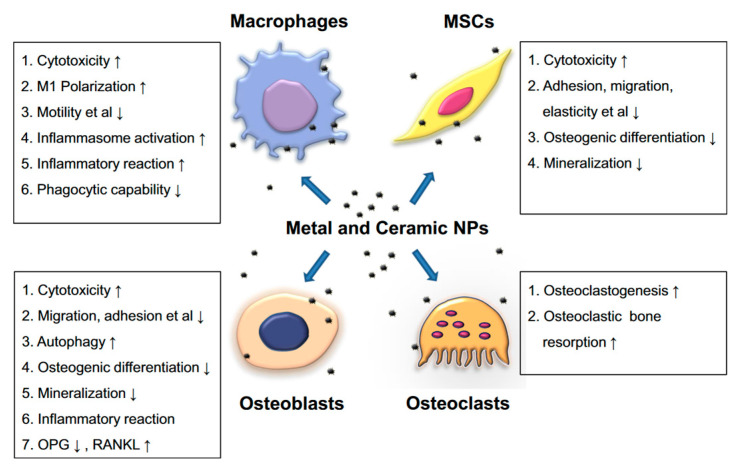
The particles’ drawbacks and negative effects on the cell functions at the implantation site [46].

Osteolysis due to metallic, ceramic, or polymeric particles represents one of the leading causes of aseptic loosening. After the knee prosthesis is introduced into the human body, it becomes a particle source due to wear and corrosion. The resulting particles infiltrate the patient’s circulatory system, or they can agglomerate in neighboring tissues such as bone or bone marrow. They have a dangerous potential to interact with osteoblasts, preosteoblasts, mesenchymal stem cells (MSCs), macrophages, osteoclasts, and fibroblasts [43]. Figure 1 presents the adverse effect of the particles against the functions of the cells placed in the implant vicinity. The pro-inflammatory effect of the macrophages and osteoblasts has an important contribution to a chronic inflammatory environment near the implant and osteoclastogenesis [44,45]. In addition, a negative influence of the osteogenic differentiation and osteoblasts or MSCs mineralization was put in evidence in the literature [46].

Retrieval analysis of well-functioning and failed implants is of great importance in the orthopedic biomaterials field because surgical technique, implant design, and material quality can be improved in order to repair joints affected by diseases or trauma [14,47]. The standard ASTM F561-97 recognizes the importance of this analysis and concludes that two factors are met to obtain a successful implant integration [48]. The first one is related to the material, and the second one is biologically dependent [49]. Some authors identified the existence of a dynamic interface placed at the implant-tissue boundary [50]. Osseointegration was defined in [51] as a direct structural and functional link between the load stress implant and vital bone. The main types of cells that act in bone healing and formation are the osteoblasts, that form bone at a rate of 0.17 mm^3^/day; osteocytes, which are considered an important stress detector for bone; and osteoclasts, that resorb bone at a rate of 100 μm/day [50]. Three different modalities of bone response were identified. The first one is called fibrointegration, which appears in a case of an extent trauma and consists of fibrous tissue apparition around the prosthesis. Then if new blood vessels are not formed, dead bone can be seen in the implant vicinity, and if all the conditions are met, normal bone can be observed in the region of the prosthesis. This last response is called osseointegration. Three types of osseointegration are present: biointegration, mechanical integration, and chemical integration. Biointegration is possible when active biomaterials that link collagen fibers to the bone, such as calcium phosphate coatings, are used. Mechanical integration is considered when the bone is connected to the implant with the help of screws, vents, or cement. Chemical integration is obtained when chemical or physical bonds such as covalent bonds, hydrogen bonds, or van der Waals forces appear. The main factors identified in the literature [52] that influence the osteointegration process are surgical technique, the biocompatibility of the materials, implant design and surface, quality of the patient bone, and loading conditions of the prosthesis. The materials can be classified according to their biocompatibility as biotolerant, bioinert, and bioactive. Regarding the stress conditions, it was noticed that implant design and geometry have a strong influence on the loads applied and can be a critical factor in the retrieval analysis. Additionally, aspects linked to the surgical technique, such as implant alignment and surgical insert point of the prosthesis, are important in stress management. The surface state influences the bonds between the bone and the implant, and critical factors such as coatings or roughness must be considered for good osseointegration.

There are many studies regarding the retrieval analysis of UHMWPE tibial inserts, and the main failure reasons are due to oxidation and wear phenomena. Liza et al. [53] investigated the failure analysis of a UHMWPE tibial insert from Apollo^®^ Total Knee System that was removed after 10 years of service. Using different testing methods such as scanning electron microscopy (SEM), infinite focus microscope (IFM) coupled with energy disperse spectroscopy (EDS), Fourier-transform spectroscopy (FTIR), and gel permeation chromatography (GPC), they concluded that the UHMWPE material exhibited high oxidation and wear degradation. Moreover, delamination, folding, pitting, and scratching were predominant. The retrieved sample had a reduced molecular weight contributing to the implant failure. Kurz et al. [54,55] and Edidin et al. [56,57] presented a mechanical test strategy adapted to UHMWPE tibial inserts called the small punch test. They have prepared small, thin disks indented by a hemispherical punch, determining a biaxial tension state. Mechanical quantities such as Young’s modulus, ultimate load, ultimate displacement, and work to failure were measured and correlated with the in vivo service. Cho et al. [58] showed that microscopic surface asperities on the knee prosthesis’s metallic femoral components have an important role in increasing the wear process of UHMWPE tibial inserts. Medel et al. [59] collected 119 tibial inserts, from which 29 were gamma sterilized in normal atmospheric conditions and 90 were conventional gamma sterilized using nitrogen atmosphere. They have found a direct link between in vivo oxidation and fatigue deterioration. It was concluded that oxidation strongly influences delamination since pitting damage is not increased by this phenomenon. Berry et al. [60] made a similar study on 132 gamma-air and 174 gamma-inert-sterilized UHMWPE tibial inserts. They noticed that gamma-inert-sterilized tibial inserts reached the critical oxidation index value at an average of 13 years after the primary surgery. Cerquiglini et al. [61] studied the new antioxidant polyethylene retrieval analysis. They used 24 Press Fit Condylar (PFC) Sigma (DePuy) and 17 Attune Knee System (DePuy) with fixed bearing and rotating platform design. A higher value of the Hood score was found in the case of Attune tibial inserts on the backside surface. Material properties’ modifications were reported due to implant design and potentially reduced implant performance was foreseen. Raman spectroscopy was used by Tone et al. [62] to analyze the wear rate and creep of e-beam-sterilized conventional UHMWPE tibial insert. The main conclusion is that the body mass index (BMI) greatly influences implant failure, and a separation of the creep and wear components of thickness reduction was made. There are few recent papers regarding failure analysis of primary cemented Zimmer Biomet knee implants. Our study fulfills this research and sustains the conclusions of Granquist et al. [63], that found a minimum 5-year survival time for total hip or knee arthroplasty. Additionally, the paper brings new information about the potential of microscopy techniques in failure explant analysis.

In the paper, we investigated four UHMWPE tibial inserts retrieved from patients from Clinical Hospital Colentina, Bucharest, Romania. We performed macrophotography, stereomicroscopy, and scanning electron microscopy investigations to establish the samples’ wear grade and to correlate it with the Hood index computation. We checked the chemical composition, crystallinity grade, and oxidation index through Fourier-transform infrared spectroscopy (FTIR). We analyzed the material’s mechanical behavior based on the small punch test.

## 2. Materials and Methods

### 2.1. Experimental Samples: Patients Data and Associated Pathologies

The investigated UHMWPE tibial inserts were retrieved from four patients from Clinical Hospital Colentina, Bucharest, Romania. Similarly, failed implants were selected for comparative analysis and reference after different implant life spans. Happily, there are just a few revision surgeries per year in the Orthopedy Section of the hospital mentioned above. The surgical technique was improved, and usually, the failure causes are due to UHMWPE tibial insert deterioration or improper cementation of the prostheses. In Colentina Hospital, there are implanted only cemented prostheses. A standard protocol is followed for the retrieved implants. After the implant is extracted, mandatory antibacterial and antimicrobial tests are performed. If their results are positive future material investigations are stopped and the prosthesis are destroyed. In the case of negative results, the implants undergo a gamma sterilization procedure, and other material tests are recommended. Our retrieved UHMWPE tibial inserts received from Clinical Hospital Colentina laboratories negative results for all the tested pathogens. All cases had the primary surgical intervention performed in the Orthopedy Section, with a time-to-failure period comprised between 5 and 15 years. The age span of the patients ranged between 54 and 69 years (lower than 70 years). Other criteria for patient selection were Caucasian race, non-smoking, and BMI lower than 30 (non-obese). Table 1 presents the experimental samples and patient data with knee implant failure and associated pathologies.

### 2.2. Characterization Methods

To analyze the samples’ surface morphology, in order to accurately identify their degradation modes and to compute the Hood index according to [64] macrophotography, optical and scanning electron microscopy methods were used. The macroscopic analysis was performed with a Canon EOS camera. The optical images were obtained with a Nikon SMZ1270i stereomicroscope, using NIS—Elements D software in Koehler illumination mode. The images were acquired with a Pixelink camera, which permits the sample surface visualization in real-time and the acquisition of high-quality images. The scanning electron microscopy analysis was performed based on a Philips XL 30 ESEM TMP microscope (FEI/Philips, Eindhoven, Netherlands) that has a low vacuum secondary electron detector (SE) and a backscattered electrons detector (BSE). The images were processed via ImageJ 1.50 software (Bethesda, MD, USA).

The oxidation index and the crystallinity percent of the retrieved tibial inserts were computed based on experimental measurements made with a JASCO 6200 FT-IR Spectrometer with a Golden Gate-type attenuated total reflection device (ATR) (Tokyo, Japan). This device has a measurement wavenumber range between 7800 cm^−1^ and 350 cm^−1^ and executes accurate measurements based on a 28° Michelson interferometer Corner cube mirror with DSP control, auto-alignment mechanism, and sealed structure. The measurements were obtained and processed via Spectra Measurements and Spectra Analysis software.

Mechanical characterization was carried out using TTS 190 equipment from Unconventional Testing Machines based on the small punch test method following SIST EN 10371:2021 [65]. The cylindrical samples with a diameter of 8 mm and a thickness of 0.5 mm were cut with the GE76 tool for specimen manufacturing. For each retrieved UHMWPE tibial insert, two samples were perpendicularly collected from the deteriorated articulating surface of each condyle. The mechanical tests were made in equibiaxial tension at a rate of 0.5 mm/min. Initial stiffness, initial peak load, ultimate load, ultimate displacement, and work to failure were determined from the load-displacement dependencies described in [54,55].

Analyzing the wear mechanisms that lead to knee implant failure and investigating the clinical conditions and techniques that influence this phenomenon represents an important step in designing and manufacturing new knee implants. These new models must exhibit a low wear rate and high life expectancy.

The most common failure cause is due to polyethylene particles that result from the tibial insert wear process [66,67]. Generally, the wear phenomenon of the articular components is very complex, including different mechanisms [68,69]. Mechanical, physical, and chemical interactions can be simultaneously observed. Delamination and adhesive wear are the most pronounced wear effects [70,71].

Hood et al. [64] identified, based on personal experience and literature review [72,73], seven deterioration modes of surface degradation in UHMWPE. The first mode consists of burnishing (M1), which is characterized by the apparition of highly polished zones that can be visualized through optical microscopy [74]. Scratching (M2) is described through the presence of aligned lines placed in an anteroposterior direction and included in the deteriorated area. Another surface deterioration mode is described as punctual defects (M3), denoted as pitting or crater formation. It is characterized by the apparition of irregularly shaped depressions with 2–3 mm across and 1–2 mm deep. Surface deformation (M4) is a term used to describe a permanent deformation present on or around the articular surface due to UHMWPE’s flow and/or creep. Delamination (M5) consists of wide zones on the tibial component surface, in which a large sheet of material was removed [75]. These zones are usually placed parallel to the articulating surface. Debris (M6) is a general term that includes pressed particles entirely or partially included into the surface, impacting the polishing effect. This deterioration mode has as main cause the polymethylmethacrylate (PMMA) cement or even human bone damage [76]. Abrasion or adhesive wear affected zone (M7) is usually described in the literature as having a tufted or shredded appearance. It is due to prolonged contact and mechanical stresses between the implant and PMMA cement or knee bones [77].

**Figure 2:**
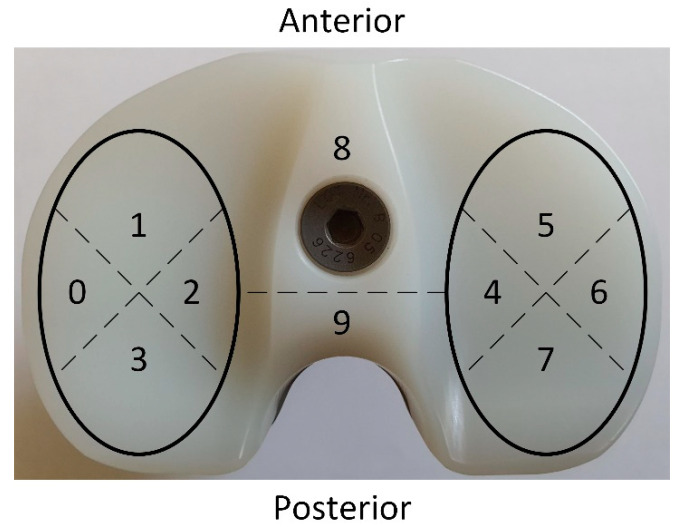
The partition system proposed by Hood et al. [64] for the quantification of deterioration modes in the case of UHMWPE tibial insert of knee prostheses.

In order to establish the grade of the deterioration process and its precise localization on the tibial component insert, we have adopted the ten-section model proposed by Hood et al. [64] (Figure 2). The severity of each deterioration mode was subjectively graded, starting with 0 and ending with 3. Grade 0 means that the defect is absent; grade 1 is correlated with the defect apparition on less than 10% of the surface since grades 2 and 3 are attributed when the deterioration mode widening is present in 10–50% and over 50% of the section surface [64].

## 3. Results and Discussion

### 3.1. Surface Deterioration Mechanisms

Following macrophotography analysis, a first assessment of the UHMWPE degradation modes was performed. The computation of the Hood index for each sample was finalized after a comparison between macrophotography, stereomicroscopy, and scanning electron microscopy results was performed.

**Figure 3:**
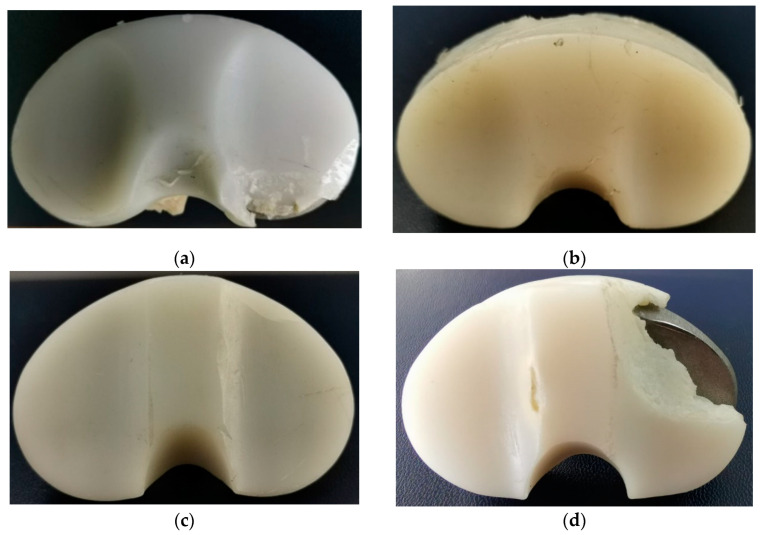
Macrophotographs of the UHMWPE tibial inserts retrieved from patients of Clinical Hospital Colentina. (**a**) IT1 sample—M2, M4, M5, M7 deterioration modes; (**b**) IT2 sample—M2, M3, M5, M7 deterioration modes; (**c**) IT3 sample—M1, M2, M3, M7 deterioration modes; (**d**) IT4 sample—M1, M2, M3, M4, M5, M7.

In Figure 3, the four investigated samples are presented, denoted as IT1, IT2, IT3, and IT4.

**Figure 4:**
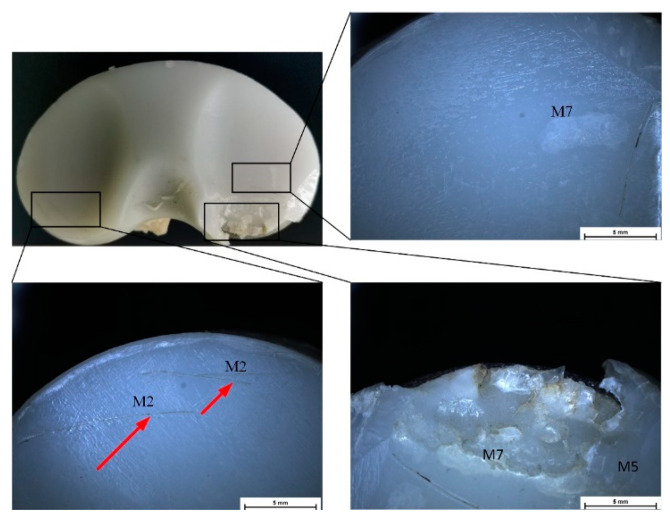
Stereomicroscopy images obtained in the case of IT1 sample.

**Figure 5:**
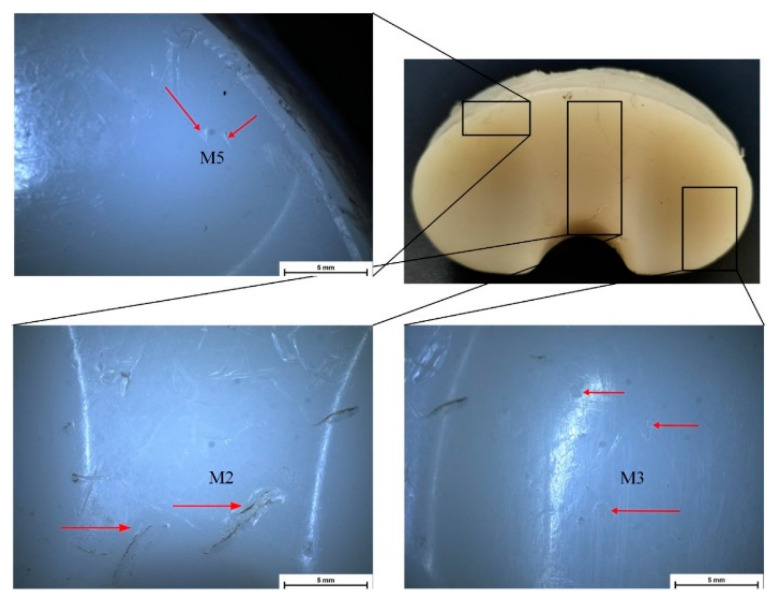
Stereomicroscopy images obtained in the case of IT2 sample.

**Figure 6:**
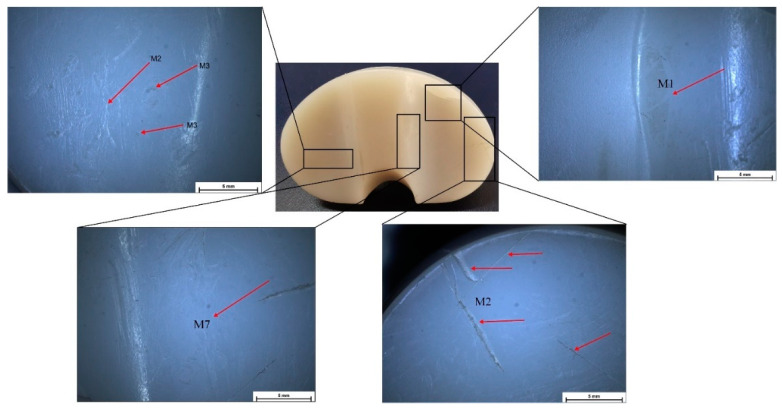
Stereomicroscopy images obtained in the case of IT3 sample.

**Figure 7:**
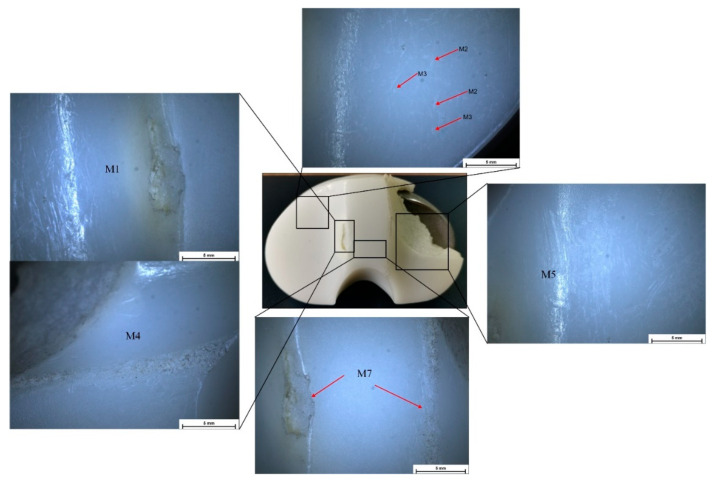
Stereomicroscopy images obtained in the case of IT4 sample.

Stereomicroscopy is a much more accurate method for deterioration modes’ identification because some defects are impossible to identify in macrophotographs or with the naked eye. In Figure 4, Figure 5, Figure 6 and Figure 7, some defect images are presented, obtained with a Nikon SMZ1270i stereomicroscope. In the case of the IT1 sample, the M2, M7, and M5 deterioration modes were put in evidence. For IT2, the M2, M3, and M5 existence confirmed the macrophotography conclusion. The tibial insert IT3 exhibited M1, M2, M3, and M7 defects, and the IT4 sample, which is the most damaged, presented M1, M2, M3, M4, M5, and M7 surface degradation mechanisms.

**Figure 8:**
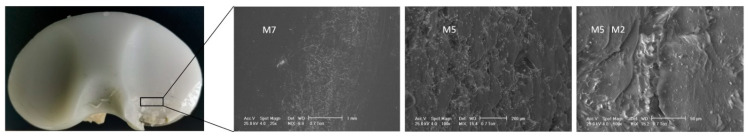
SEM images obtained for IT1 sample.

**Figure 9:**
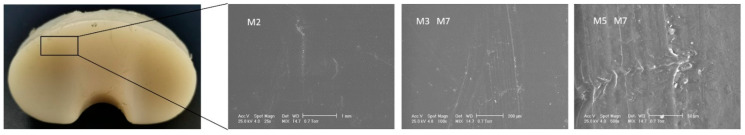
SEM images obtained for IT2 sample.

**Figure 10:**
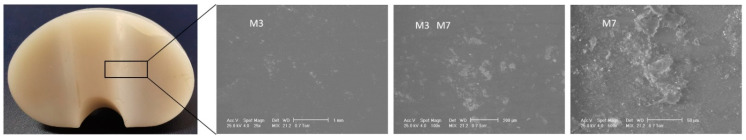
SEM images obtained for IT3 sample.

**Figure 11:**
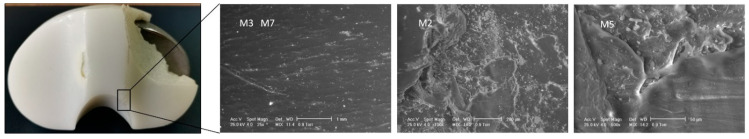
SEM images obtained for IT4 sample.

A Philips XL 30 ESEM TMP microscope was used to characterize the surface morphology of the UHMWPE tibial inserts. The results obtained through macrophotography and stereomicroscopy were reinforced and confirmed by the scanning electron microscope images. In the case of IT1, the presence of delamination (M5), scratching (M2), and abrasive wear (M7) was clearly put in evidence (Figure 8). IT2 tibial insert exhibited scratching (M2), punctiform defects (M3), delamination (M5), and abrasive wear (M7) (Figure 9). For the IT3 sample, SEM images (Figure 10) showed punctiform defects existence (M3) and abrasive wear presence (M7), although following the results obtained through previous methods, we have to take into consideration when we compute the Hood index to also account for the scratching (M2) and burnishing (M1) modes. It can be noticed that the M1 deterioration mode cannot be easily observed through SEM investigations. Scanning electron microscopy confirmed that the IT4 component is the most damaged one, its surface exhibiting punctiform defects (M3), abrasive wear (M7), scratching (M2), and delamination (M5) (Figure 11). Through stereomicroscopy and macrophotography, we put additional evidence of the existence of burnishing (M1) and surface deformation (M4) modes.

Through SEM microscopy, the retrieved tibial component surface can be investigated at a micrometric level. This type of analysis is very accurate. However, some deformation modes, such as burnishing and surface deformation, cannot be observed. Consequently, this method must always be combined with macrophotography and/or stereomicroscopy to obtain a precise result and an exact computation of the Hood index. From the SEM images, it can be concluded that debris of PMMA cement or bone are absent. This deterioration mode can be put in evidence only through scanning electron microscopy because, in the case of the macroscopic observations, it is impossible to be observed.

In Table 2 are presented the Hood index values computed based on experimental observations for the four retrieved tibial inserts.

From Table 2, it can be noticed that the tibial insert surface deterioration varies from patient to patient, being influenced by different factors such as insert material, the shape of the investigated component, patient height and weight, the articular range of motions during daily activities, and surgical technique regarding the implant alignment and viable soft tissue quantity. Many of the variables mentioned above cannot be controlled, so developing new high-quality biomaterials with an increased wear resistance is seen as a worldwide necessity.

Usually, the polyethylene insert deteriorates due to lower interactions between polymer chains by comparing it with those determined in the case of atoms’ interactions in metallic or ceramic materials [61,74]. We have found for our retrieved UHMWPE inserts that delamination (M5) is the most important deterioration mode that is in good agreement with the literature [78,79]. Delamination is found for three samples, being much more pronounced in the case of the IT4 sample. We can observe that M5 is present in four sections and in three zones, to which we have attributed the maximum number of points. Burnishing (M1) is a surface deterioration mode that can be seen only in the case of IT3 and IT4 samples. Many scratching (M2) defects can be observed for all the samples, and their position varies from sample to sample. The most pronounced scratching is noticed for the IT3 sample in sections six and seven. Punctiform defects (M3) are present for IT2, IT3, and IT4 samples, and surface deformation (M4) is visible for IT1 and IT4 samples, being more accentuated in the case of the last sample. Polyethylene abrasive wear (M7) is a deterioration mode that was observed for all the samples with maximum allocated points for the IT4 sample in three sections. Cement or bone debris (M6) are absent because SEM micrographs did not show their existence.

By analyzing the Hood index values presented in Table 2, we can conclude that the IT4 sample is the most damaged one. In this case, we detected five deterioration modes with a Hood score of 32. Samples IT1 and IT2 have a Hood index of 9 and IT3 of 10.

Nowadays, it is estimated that all total knee implants have a survival rate of about 95% after 10 years post-surgical intervention, 88.7% after 15 years, and 82% after 25 years [80]. The life span of polyethylene components is limited to a maximum of 20 years [81,82]. Thus, the main failure reason in TKA is exclusively due to UHMWPE particles that appear due to wear and oxidation mechanisms [83,84]. This phenomenon is seen in the young and active population, and many cases of premature implant failure due to polymer particle-induced osteolysis are reported in the literature [85,86].

### 3.2. Oxidative Degradation Analysis

Oxidative degradation of the UHMWPE represents one of the main failure reasons for total knee implants [62,87]. This process can estimate the material’s life expectancy in accordance with international standards [88,89]. The average life span of UHMWPE tibial inserts is estimated at 15–20 years, being strongly influenced by its deterioration grade [67]. Fourier-transform infrared spectroscopy (FTIR) represents the experimental method of choice for the oxidation process identification in polymeric materials.

The oxidation mechanism of short-chain hydrocarbons such as UHMWPE is made according to the Bolland cycle [88]. FTIR analysis can identify all the functional groups that correspond to the Bolland cycle and offers information regarding the chemical bond vibrations. In order to accurately estimate the oxidation grade of the samples, we have introduced a control specimen that was never used in the patient body.

The oxidation process of UHMWPE can be quantified based on FTIR experiments through a standard protocol [89,90]. The dimensionless oxidation index (OI) is computed based on a normalization procedure that uses the height or the area of the peak associated with the carbonyl vibration (C=O) placed at a wavenumber of about 1720 cm^−1^. There are two standard protocols described, which use the normalization of the quantities mentioned above with the peak height or area placed at 1370 cm^−1^ or 2022 cm^−1^ [90,91]. These two reference peaks are associated with UHMWPE material’s crystalline or amorphous phase. As the polyethylene oxidates after the irradiation manufacturing step, the broken molecular chains recrystallize, and as a direct consequence, the mass density and crystallinity grade increase. It can be concluded that the material’s crystalline and amorphous phases evolve since the polymer degrades and the absorption peaks associated with these states become modified.

The spectra were determined in the range of 4000 cm^−1^ to 600 cm^−1^ at a resolution of 4 cm^−1^ after 64 scans. Firstly, a background measurement was performed for each sample to eliminate environmental influences such as carbon dioxide content or air humidity. We cut small parts from the control and retrieved tibial inserts with a plane surface because in our measurements it was involved an attenuated total reflectance accessory (ATR) that permits spectra measurements without additional surface treatments.

**Figure 12:**
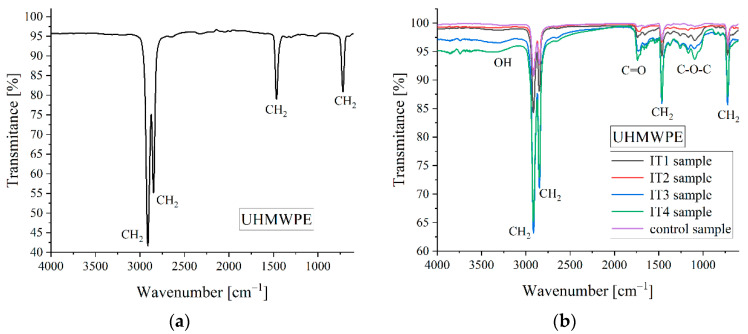
Spectra for the UHMWPE. (**a**) Standard ideal FTIR spectrum for unoxidized UHMWPE sample [92]; (**b**) FTIR spectra for the control and retrieved UHMWPE samples.

In Figure 12, the standard ideal spectrum for UHMWPE [92] and the spectra obtained for control and retrieved samples are presented.

In Table 3, the wavenumbers that correspond to the peak identified in the FTIR spectra and the reference wavenumber used for the oxidation index computation are presented.

The oxidation index was calculated as the ratio between the area of the carbonyl peak (A_C=O_), determined for each sample, and the area that corresponds to the reference peak placed at about 1370 cm^−1^ (A_ref_) (Equation (1)).

OI = A_C=O_/A_ref_, (1)

The areas mentioned above were computed with the Spectra Analysis software. The obtained results are shown in Table 4.

It can be concluded that the control sample has the lowest oxidation index since the IT4 sample has the highest value. This fact is in good accordance with the Hood index computation because this specimen had the most pronounced surface deterioration. Although the IT2 sample exhibited a reduced Hood index value, its oxidation index is high. This fact shows the importance of FTIR analysis because, in some cases, the overall material deterioration cannot be identified only with visual investigation methods. The samples IT1 and IT3 have similar oxidation and Hood index values.

The FTIR analysis permits the crystallinity index computation based on an empirical formula developed by Zerbi et al. [93] that provides the amorphous phase content according to Equation (2).

X = (|1 − I_a_/I_b_|/1.233)/(1 + I_a_/I_b_) × 100, (2)

where I_a_ and I_b_ correspond to the absorption intensities of the peaks localized at 1474 cm^−1^ and 1464 cm^−1^. The constant value of 1.233 is given by a relationship between peaks that characterize the crystalline phase of the material. Based on the Spectra Measurements software and deconvolution function, which gives us an adequate tool for separating of the overlap peaks from the FTIR spectra, we have identified the height of the peaks mentioned above and computed the amorphous substance content for each sample (Table 5).

Low content of amorphous substance is equivalent to a high crystallinity grade. It is well known that the crystallinity index increases while the UHMWPE degrades due to the oxidation process. It can be noticed that the IT4 sample has the lowest amorphous substance percent and the highest value of the oxidation index (Table 4). In the control sample case, we could not identify the peak placed at 1474 cm^−1^ after the deconvolution procedure, so Equation (2) cannot be applied. Otherwise, the peak at 1464 cm^−1^ was visible in the FTIR spectrum. We have concluded that this observation is in good agreement with the low value of the oxidation index.

### 3.3. Mechanical Characterization

**Figure 13:**
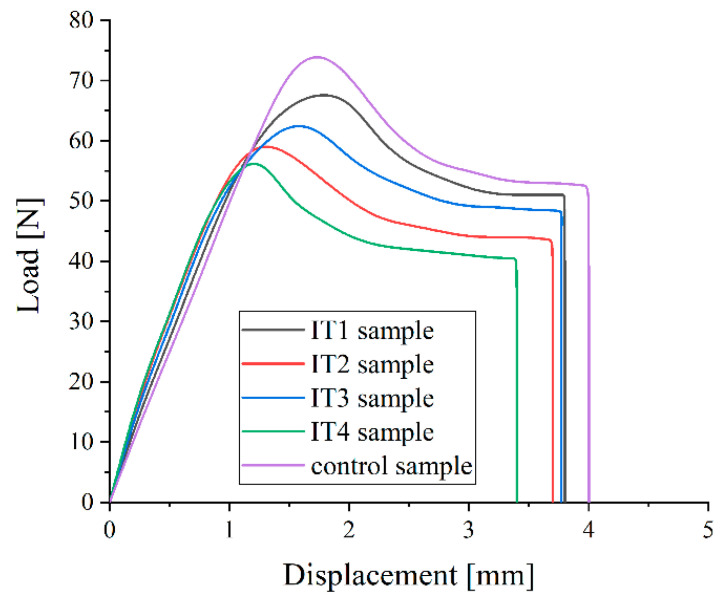
Small punch test load-displacement curves for the control and retrieved UHMWPE samples.

Following the procedure described in [54,57], the initial stiffness is proportional to the UHMWPE Young’s modulus (E). The initial peak load represents the maximum load value extracted from the load-displacement curve. The ultimate load and displacement are the last point coordinates determined before the material fracture. The work to failure is computed as the area placed under the load-displacement variation, and it is a quantity related to the material toughness [39]. In Figure 13, the load-displacement variations obtained for the samples prepared from IT1, IT2, IT3, IT4, and control UHMWPE tibial inserts are presented.

A correlation between the oxidation index of the material and the mechanical properties determined through the small punch test can be noticed. Young’s modulus increases directly proportional to the oxidation index. The initial peak load, ultimate load, and ultimate displacement decrease when the oxidation state of the UHMWPE sample increases. From Figure 13 it can be observed that all the tested samples exhibit the classical load-displacement curve shape that shows a typical behavior of UHMWPE [54,57]. Above the elastic region, the dependence load versus displacement presents an initial peak load that appears because of sample bending. This peak is followed by a stretching phase. The ultimate strength and toughness decrease is usually associated with high surface deterioration. The toughness has an important contribution to reducing the fatigue resistance of UHMWPE tibial inserts after gamma irradiation [54]. The control sample has the lowest value of Young’s modulus with an average of 987 MPa and the highest initial peak load, ultimate load, and displacement values. The sample IT4 has the highest elasticity modulus (average value of 1300 MPa) and the lowest values of other mechanical parameters, which is explained by a high value of the oxidation index.

In Table 6, the average values and standard deviations for the mechanical quantities are presented.

As future directions and stabilization against oxidation phenomenon, the crosslinking process must be carefully controlled and optimized without reducing the material’s mechanical properties and with increased wear resistance. Moreover, the use of vitamin E prevents the oxidation that is associated with free radicals. Today the ASTM Standard F 2695 2007 [94] permits the development of crosslinked UHMWPE inserts, which have added vitamin E before the irradiation procedure, during extrusion or molding steps, or using diffusion after the irradiation. The main drawbacks consist of the fact that the crosslinking is suppressed to a minimum amount during irradiation and the difficulty in controlling the concentration and distribution of vitamin E. Other methods such as surface texturing [95], surface coating, ion beam surface modification, photolithography and nanoimprint lithography [96], or the use of reinforcing particles that are combined with UHMWPE to increase the mechanical properties and to reduce the wear rates such as micro and nano ZnO particle [97], zeolite particles [98], Zr particles [99], and carbon-based materials [96] were proposed in the literature.

## 4. Conclusions

UHMWPE represents the golden standard in knee arthroplasty for the tibial insert manufacturers. In the paper, we found a good correlation between microscopical methods used for wear analysis, SEM results adding some valuable information regarding the absence of cement or bone debris in the retrieved tibial components. We have identified all surface deterioration mechanisms, such as burnishing, scratching, punctiform defects, surface deformation, delamination, and abrasive wear.

The deterioration mechanism in UHMWPE tibial inserts is associated with the oxidation phenomenon due to the corrosive medium inside the human body [89,93]. We have computed UHMWPE’s oxidation index and amorphous content and found a direct correlation between them. The mechanical properties were investigated, and it was concluded that the oxidation phenomenon is directly linked to a deterioration of the UHMWPE tibial insert mechanical properties. Young’s modulus increased in the case of samples with high oxidation index values since the ultimate load and displacement decreased.

Retrieval analysis provides the specialists with valuable information to improve the biomaterial properties and, in some cases, modify the prosthesis design. We have concluded that all the retrieved samples exhibited surface deterioration and oxidation of the material, and the main reason for implant failure was osteolysis due to UHMWPE particle emission.

As for future directions and stabilization against the oxidation phenomenon, the crosslinking process must be carefully controlled and optimized without reducing the material’s mechanical properties and with increased wear resistance.

## Figures and Tables

**Table 1:** Experimental samples and statistics data on patients with knee implant failure.

UHMWPE Tibial Insert Data				Patient Clinical Data					
Sample ID	Hospital	Implant Life Span [Years]	Producer	Patient Age [Years]	Sex	Weight [kg]/Height [m]	KS		Pathologies
							Preoperative	Postoperative	
IT1	Colentina	5	Zimmer	54	M	75/1.7	55	85	Diabetes, myelitis, vascular stroke, cervical spondylosis
IT2	Colentina	10	Zimmer	62	F	60/1.63	63	90	Diabetes, osteoporosis, cardiac insufficiency
IT3	Colentina	15	Zimmer	69	M	85/1.73	71	94	Schizophrenia
IT4	Colentina	10	Zimmer	65	F	70/1.6	73	96	Cervical spondylotic myelopathy, osteoporosis

M: male, F: female, KS: Knee Society Knee Score.

**Table 2:** Surface deterioration modes and Hood index computation for the retrieved UHMWPE tibial inserts.

Sample ID	Deterioration Mode	M1	M2	M3	M4	M5	M6	M7
IT1	Zone	-	3, 7	-	6	0, 7, 8, 9	-	7
	Grade	0	1, 1	0	1	1, 2, 1, 1	0	1
	Hood score	9						
IT2	Zone	-	1, 3, 8, 9	1, 6, 7	-	1	-	1
	Grade	0	1, 1, 1, 1	1, 1, 1	0	1	0	1
	Hood score	9						
IT3	Zone	5	3, 4, 5, 6, 7	3, 4	-	-	-	4
	Grade	1	1, 1, 1, 2, 2	1, 1	0	0	0	1
	Hood score	10						
IT4	Zone	2, 3	1, 9	1,9	2	2, 5, 6, 7, 9	-	4, 5, 6, 9
	Grade	1, 1	1, 1	2, 1	2	2, 3, 3, 3, 1	0	3, 3, 3, 2
	Hood score	32						

**Table 3:** Wavenumbers associated with the peaks identified in the FTIR spectra for the five tested samples.

Sample ID	C-H Asymmetric Stretch in CH_2_ [cm^−1^]	C-H Symmetric Stretch in CH_2_ [cm^−1^]	CH_2_ Bend [cm^−1^]	CH_2_ Bend [cm^−1^]	C=O Stretch [cm^−1^]	C-O-C Stretch [cm^−1^]	OH Stretch [cm^−1^]	Reference of 1370 cm^−1^ Used for OI Computation [cm^−1^]
IT1	2914.8	2845.4	1465	720.2	1738	1166	3300	1369
IT2	2912.9	2846	1465	720	1720	1167	3300	1365
IT3	2913	2845	1464.7	721	1728	1166	3300	1369
IT4	2913.9	2844	1464.6	720	1735	1165	3300	1369.2
Control	2914	2845	1463.7	720	1738	1160	-	1366

**Table 4:** Oxidation index computation for retrieved and control samples characterized based on FTIR spectroscopy.

Sample ID	A_C=O_ [cm^2^]	A_ref_ [cm^2^]	OI
IT1	63.44	12.04	5.26
IT2	45.219	4.683	9.66
IT3	118.079	17.11	6.9
IT4	185.91	18.99	9.78
Control	7.67	5.76	1.33

**Table 5:** Amorphous substance content in the case of the retrieved and control samples characterized based on FTIR spectroscopy.

Sample ID	X [%]
IT1	56
IT2	62
IT3	67
IT4	15
Control	-

**Table 6:** Mechanical properties of the retrieved and control samples after the small punch test.

Sample ID	Young’s Modulus [MPa]	Initial Peak Load [N]	Ultimate Load [N]	Ultimate Displacement [mm]	Work to Failure [mJ]
IT1	1100 ± 175	68 ± 1.5	51 ± 2.6	3.8 ± 0.07	188.92 ± 6
IT2	1267± 170	60 ± 3.1	44 ± 4.3	3.7 ± 0.03	163 ± 3
IT3	1160 ± 180	63 ± 1.8	48 ± 2.9	3.77 ± 0.2	178.4 ± 6
IT4	1300 ± 180	58 ± 10	40.5 ± 3	3.4 ± 0.04	139.88 ± 2
Control	987 ± 175	75 ± 2.5	52 ± 1.8	4 ± 0.5	206.06 ± 5

## Data Availability

Not applicable.
